# Apigenin exerts protective effect and restores ovarian function in dehydroepiandrosterone induced polycystic ovary syndrome rats: a biochemical and histological analysis

**DOI:** 10.1080/07853890.2022.2034933

**Published:** 2022-02-12

**Authors:** Fangxin Peng, Yichuan Hu, Shu Peng, Ni Zeng, Lei Shi

**Affiliations:** a Department of Reproductive Medicine, Maternal and Child Health Hospital of HuBei Province, Hubei, China; b Department of Anaesthesiology, Wuhan Union Hospital, Tongji Medical College, Huazhong University of Science and Technology, Hubei, China

**Keywords:** Apigenin, polycystic ovarian syndrome, lipid profile, anti-oxidant

## Abstract

**Background:**

Polycystic ovarian syndrome (PCOS) is one of the major causes encouraging the elevation of androgens, obesity along with menstrual complications. Here we study the effect of Apigenin in rat model of polycystic ovarian syndrome.

**Methods:**

Female Sprague Dawley (SD) rats were treated with Dehydroepiandrosterone (DHEA) (6 mg/100g) opting the post-pubertal approach for developing rat model of polycystic ovarian syndrome, Metformin was used as standard. The treatments were given for 21 days along with coloproctological analysis. After the treatment regimen, the biochemical analysis was carried in plasma samples, whereas the ovaries were submitted for histopathological analysis.

**Results:**

The treatment of DHEA resulted in disturbed lipid profile and anti-oxidant status along with increased weight, ovarian diameter and cysts in rats confirming the development of PCOS. However, treatment of Apigenin showed ameliorative effect by improving the lipid profile and anti-oxidant status, the treatment also normalised the body weight, reduced ovarian diameter, cysts and restored the healthy follicles compared to control rats. The treatment of Apigenin also suppressed the levels of oestradiol and testosterone compared to control group, also, levels of progesterone were increased in Apigenin treated group of rats. The treatment of Apigenin suppressed the levels of inflammatory cytokines TNF-α and IL-6. It was observed that the effect of Apigenin were to some extent parallel to standard drug Metformin.

**Conclusion:**

The findings confirmed that Apigenin ameliorates the disturbed hormonal levels, lipid profile and antioxidant status in PCOS rats.

## 1. Background

Polycystic ovarian syndrome (PCOS) is a metabolic disorder in premenopausal women involving reproductive and endocrine system. The rate of incidence variers from 5 to 20%, the rate goes higher in indigenous women suggesting role of genes in its pathophysiology [1]. The diagnosing features for PCOS as per the Rotterdam’s criteria include ovarian cysts, dysfunction of ovulatory system and hyperandrogenism [2]. Chronic PCOS leads to cardiovascular disorders, obesity, diabetes, carcinoma of endometrium and amenorrhea/oligomenorrhea [3–5].

The PCOS condition is accompanied by abundance of androgen as a result of insulin resistance, obesity or by androgen secreting neoplasms [1]. The symptoms of PCOS are acanthosis nigricans, acne, hirsutism and seborrhoea, the condition is also associated with hyperinsulinemia and hyperandrogenism [6]. Along with luteinising hormone (LH) insulin creates hyperactive androgenic state as a result of decreased hepatic fabrication of sex hormone binding globulin and hyperthecosis, which induces the anovulatory mechanisms [1].

The gonadotropin releasing hormone is one of the main regulators of the reproductive system which is responsible for initiating the release and synthesis of luteinizing hormone and follicle stimulating hormone in the pituitary gland [7]. The gonadotropin releasing hormone involves two receptors belonging to G-protein coupled receptors family [7]. The mitogen activated protein kinase (MAPK) is crucial link between transfer of signals from the cell surface to the nucleus thus regulating the transcription of gonadotropin. In addition to this the gonadotropin releasing hormone is responsible for activation of ERK mainly by phosphorylation of Raf1 [7].

The treatment of PCOS has no specific medication but repurposing of existing molecules is done. Metformin (MTF) is a thiazolidinedione derivative; it increases insulin sensitivity and is an agent of choice for treating PCOS [8]. MTF is an anti-hyperglycaemic belonging to bi-guanine class of oral anti-diabetic drugs [9]. MTF works by improving the insulin sensitivity, blocks the production of hepatic glucose and suppresses the synthesis of androgen by ovarian cells [10,11]. In addition to its therapeutic benefits, MTF has side effects which include renal insufficiency, acidosis and gastro-intestinal disturbances [9].

The condition of PCOS is found to be associated with imbalance of oxidant and anti-oxidant system [12,13]. Hence, involvement of oxidative stress in PCOS cannot be ruled out as it is associated with other metabolic diseases such as diabetes, cardiovascular disorders, obesity and atherogenesis [14].

Oxidative stress is defined to be as an imbalance between production and scavenging of reactive oxygen species [15], it is found that an excess of generation in reactive oxygen species leading to their accumulation *in vivo* would cause damage of cells [16,17], protein [18–20], and lipids [21]. Oxidative stress is reported to be involved in pathogenesis of PCOS as the patients of PCOS have been found to show increased oxidative stress compared to normal [22]. Oxidative stress has been found to cause abnormal proliferation of endometrial cells also called as hyperplasia of ovary which further leads to PCOS, decreased fertility and endometriosis [23]. In addition to this oxidative stress is associated with pathological processes such as hyperandrogenemia and obesity, which are most of the times associated with PCOS.

Bioactive compounds have been used widely and successfully for treating gynaecological disorders [24,25]. Among bioactive compounds flavonoids are of major interest due to their wide range of pharmacological properties [26]. Apigenin (APG) is flavonoid present widely in fruits, vegetables and many Chinese herbs. The flavonoid APG is found to show anti-oxidant, anti-inflammatory and anti-cancer activities [27,28], hence APG has been used from ancient times as an important component of traditional Chinese medicine. In addition to this, APG have been reported to exert cholesterol regulating activity [29,30].

Looking into the limited treatment options for PCOS and the side effects associated with existing treatments there is an urgent need of a therapeutic agent which would be specific in treating PCOS with fewer side effects, this made us focus on APG due to its pharmacological activities. In the present study, we investigated the possible effects of APG in rat model of PCOS.

## 2. Materials and methods

### 2.1. Animals, PCOS model and experimental design

For developing the PCOS rat model we followed the procedure developed by Kim et al. [31] which reported an improved PCOS rat model. For developing the dehydroepiandrosterone (DHEA) rat model, we used Sprague Dawley dams having 8 to 10 pups which were supplied by the animal centre. The pups were housed under controlled conditions of temperature of 23 ± 2 °C with relative humidity of 65% and 12 h dark light cycle. The pups were given special chow diet composed of 4.6% fat, 21.68% protein, 6% ash and 2.5% minerals and water *ad libitum*. Prior to inducing PCOS, the experiments were approved by animal ethical review board. The inclusion criteria for the rats as per the protocol was 10% weight loss with less than 40% food intake as sham group within seven days following DHEA treatment. The PCOS was induced by post-pubertal approach as discussed earlier [31]. For the same 42-day old female rats were used as sham and for DHEA treatment. The rats were injected subcutaneously with DHEA (60 mg/kg) in sesame oil for 20 days. The rats were divided randomly into 4 groups, Group 1 control (vehicle treated, 0.5% carboxymethyl cellulose), Group 2 positive control DHEA induced PCOS rats given treatment of vehicle (0.5% carboxymethyl cellulose), Group 3 DHEA induced PCOS rats given Metformin (MTF) (20 mg/kg), Group 4 DHEA induced PCOS rats and Apigenin (20 mg/kg po) once daily [32] solubilised in 0.5% carboxymethyl cellulose. The body weight of rats belonging to all treatment groups were recorded on every fifth day, on the 22nd day of treatment the rats were sacrificed by decapitation method.

### 2.2. Collection of vaginal smear, blood and tissue sampling

The rats before submitting to treatment, vaginal smear was collected daily with the help of dropper loaded with isotonic saline solution (0.9% NaCl). The smear was submitted to haematoxylin staining for evaluating the stages with the help of microscope [33].

After 3 weeks of treatment the rats were decapitated at the dioestrus stage. The blood from the trunk region was collected and submitted to centrifugation at 5000 g for 10 min. the plasma was isolated and kept at −20 °C before it was analysed using a biochemical analyser. The ovaries were removed and washed with isotonic saline for removing excess of fat tissues. The right ovary was cryo-freezed at −80 °C for studying the levels of antioxidant enzymes whereas the left ovary was used for histological screening and was fixed using 10% formalin solution.

### 2.3. Histological and histomorphology analysis

The isolated ovaries from experimental rats were fixed in formalin 10% and were processed for histological examination, the tissues were embedded in paraffin and sections of 5 μm were prepared using a rotary microtome, the sections were placed on glass slides, 10 sections/rat were selected and studied so as to avoid repetition of follicles. The sections were submitted to haematoxylin and eosin staining. The follicle with largest diameter bearing oocyte was considered. In the sections, the thickness of granulosa layer and theca layer were recorded using the Image processing software. The classifications of follicles were done as per the earlier reports [34].

### 2.4. Follicle classification

The cystic follicles were classified as follows, the primordial follicle showing flattened layer of granulosa cells having oocyte, the primary follicles showing cuboidal layer of granulosa cells with presence of well-defined oocyte, secondary follicle showing multiple layers of granulosa along with prominent oocyte, follicular fluid and tertiary follicle showing presence of antrum

### 2.5. Assay for antioxidant enzyme activity

For studying the involvement of oxidative stress, antioxidant enzyme activity of catalase (CAT), peroxidase (POD), superoxide dismutase (SOD), glutathione reductase (GR), Reduced nicotinamide adenine dinucleotide phosphate (NADPH) and thio-barbituric acid reactive substances (TBARS) were analysed. The isolated ovary tissue (20 mg) was homogenised using a tissue homogeniser in 2 ml phosphate buffer of pH7.4 followed by centrifugation at 10,000 g for 25 min at 4 °C. The supernatant of the resultant was used for measuring all the oxidative stress markers. The CAT and GR were evaluated using Catalase assay and glutathione reductase kit respectively (Sigma Aldrich USA), POD and NADPH by peroxidase assay and NADPH assay kit respectively (Abcam USA), SOD by superoxide dismutase kit (ThermoFisher USA), TBRAS using a TBRAS assay kit (Cayman Chemical, USA). All the procedures were in accordance to supplied instructions.

### 2.6. Biochemical profile and hormonal assay

The serum glucose levels (fasting), low-density lipoprotein-cholesterol (LDL-C), high-density lipoprotein-cholesterol (HDL-C), triglycerides and total cholesterol were evaluated using respective kits (Merck, USA) using a Biochemical autoanalyzer.

The plasma levels of oestrogen, testosterone, and progesterone were studied by ELISA method using receptive kits (Sigma Aldrich USA) following the supplied instructions. Standard calibration curve method was used for analysing the levels of oestrogen, testosterone, and progesterone.

### 2.7. Elisa analysis for inflammatory cytokines

ELISA analysis of inflammatory cytokines tumour necrosis factor-α (TNF-α) and interlukin-6 (IL-6) was done using ELISA kits. The tissue homogenates of ovarian tissues of PCOS rats were analysed by ELISA for expression levels of TNF-α and IL-6 using respective ELISA kits (Sigma Aldrich USA) following the supplied instructions.

### 2.8. Statistics

The results were presented as mean ± SEM. Analysis of variance (ANOVA) followed by post hoc analysis by Tukey’s post hoc test for multiple comparisons was done. The coefficient of variation for inter-assay and intraassay were <15% and <10 respectively. The values of *p* < .05 were considered to be significant. All the analysis was done using Graph Pad Prism software version 9.

## 3. Results

### 3.1. Effect of Apigenin on oestrous cycle and body weight

The control group of rats showed normal oestrous cycle i.e. of 4 to 5 days following a sequential order pattern, whereas a disturbed pattern of oestrous cycle was seen in PCOS induced rats showing a heavy dioestrus stage. It was observed that the PCOS rats receiving treatment of APG and MTF showed refurbishment of the oestrous cycle. The study of body weight pattern at the end of study suggested that the PCOS induced rats demonstrated about 25% increase in body weight against the control group. It was observed that the treatment of APG as well as MTF do not show any notable variations in body weight compared to control rats (Table 1).

**Table 1.** Body weight, body mass index, levels of glucose, diameter of ovary, weight of ovary, diameter of ovary.

Parameters	Control	PCOS	PCOS + MTF	PCOS + Apigenin
Body weight Initial (g)	181.5 ± 1.22	181.3 ± 1.38	181.6 ± 1.65	181.55 ± 1.45
Body weight final (g)	198.65 ± 1.88	226.3 ± 2.89	195.22 ± 1.77	199.1 ± 1.59
Glucose (mg/dl)	57.55 ± 0.55	71.50 ± 0.61***	56.44 ± 0.41^###^	56.50 ± 0.51^###^
Ovary weight (mg)	3.65 ± 0.11	5.25 ± 0.23***	3.82 ± 0.15^###^	3.87 ± 0.54^###^
Ovary diameter (mm)	28.65 ± 0.85	67.44 ± 1.95***	30.1 ± 1.43^###^	31.54 ± 1.02^###^

Values are mean ± SEM, *p < .05, **p < .01, ***p < .001 compared to control, #<.05, ##*p* < .01, ###*p* < .001 compared to PCOS group.

### 3.2. Effect of Apigenin on diameter and weight of ovaries

The PCOS induced rats showed significant increased diameter and weight of ovaries compared to the control rats. However, treatment of APG and MTF resulted in significant decreased ovarian diameter as well as weight compared to PCOS induced rats (Table 1).

### 3.3. Effect of Apigenin on morphometric parameters

The PCOS rats on morphometric analysis showed presence of number of cysts of large sizes instead of secondary and tertiary follicles. The treatment of APG in PCOS induced rats resulted in decreased follicular cysts diameter which was not seen in PCOS group. The treatment of MTF however resulted in more significant decrease in diameter of follicular cysts compared to PCOS control rats (Table 2).

**Table 2.** Thickness of peripheral granulosa, theca layer of secondary, tertiary and cystic follicles.

Parameters	Control	PCOS	PCOS + MTF	PCOS + Apigenin
Secondary follicles				
Diameter (µm)	237.85 ± 3.11	273.44 ± 5.47	230.17 ± 4.55^#^	239.45 ± 4.95^#^
Thickness of granulosa (µm)	47.22 ± 1.52	51.11 ± 2.54	42.15 ± 1.05	42.95 ± 1.85
Thickness of theca (µm)	21.54 ± 1.55	22.85 ± 1.15	22.24 ± 1.03	21.55 ± 1.13
Tertiary follicles				
Diameter (µm)	423.54 ± 9.22	473.56 ± 12.14	415.35 ± 11.21	405.25 ± 10.35
Thickness of granulosa (µm)	53.24 ± 2.56	34.52 ± 2.11**	54.25 ± 1.95^##^	50.14 ± 2.56^#^
Thickness of theca (µm)	25.65 ± 2.10	22.12 ± 1.25	24.13 ± 1.33	22.95 ± 1.52
Cystic follicles				
Diameter (µm)	0	604.52 ± 8.52***	485.15 ± 13.25^###^	528.65 ± 12.55
Thickness of granulosa (µm)	0	31.25 ± 1.14***	32.56 ± 2.55***	37.14 ± 2.58***^#^
Thickness of theca (µm)	0	25.04 ± 1.51***	23.51 ± 1.14***	27.85 ± 2.44***

Values are mean ± SEM, *p < .05, **p < .01, ***p < .001 compared to control, #<.05, ##*p* < .01, ###*p* < .001 compared to PCOS group.

**Table 3.** The number of ovarian follicles (mean ± SEM).

Developing follicles	Control	PCOS	PCOS + MTF	PCOS + Apigenin
Corpus luteum	3.55 ± 0.25	1.65 ± 0.10***	3.61 ± 0.21^###^	3.45 ± 0.25^###^
Atretic Follicle	2.83 ± 0.15	7.95 ± 0.92***	3.31 ± 0.25^###^	3.02 ± 0.23^###^
Cystic Follicle	0	11.42 ± 0.52***	1.61 ± 0.21^###^	1.63 ± 0.33^###^
Graffian	1.02 ± 0.02	0.17 ± 0.21	0.73 ± 0.16	0.86 ± 0.21
Primodial	7.86 ± 0.36	7.35 ± 0.10	8.12 ± 0.12	7.88 ± 0.15
Primary	6.83 ± 0.25	5.53 ± 0.21	6.89 ± 0.55^#^	6.93 ± 0.12^#^
Secondary	2.05 ± 0.1	0.63 ± 0.03*	2.12 ± 0.25^##^	2.01 ± 0.44^#^
Tertiary	1.63 ± 0.25	0.17 ± 0.01***	1.32 ± 0.18^##^	1.29 ± 0.20^##^
Tertiary	1.63 ± 0.21	0.17 ± 0.13	1.31 ± 0.20	1.27 ± 0.19

Values are mean ± SEM, *p < .05, **p < .01, ***p < .001 compared to control, #<.05, ##*p* < .01, ###*p* < .001 compared to PCOS group.

### 3.4. Effect of Apigenin on the thickness of peripheral granulosa and theca layer

**Figure 1.**
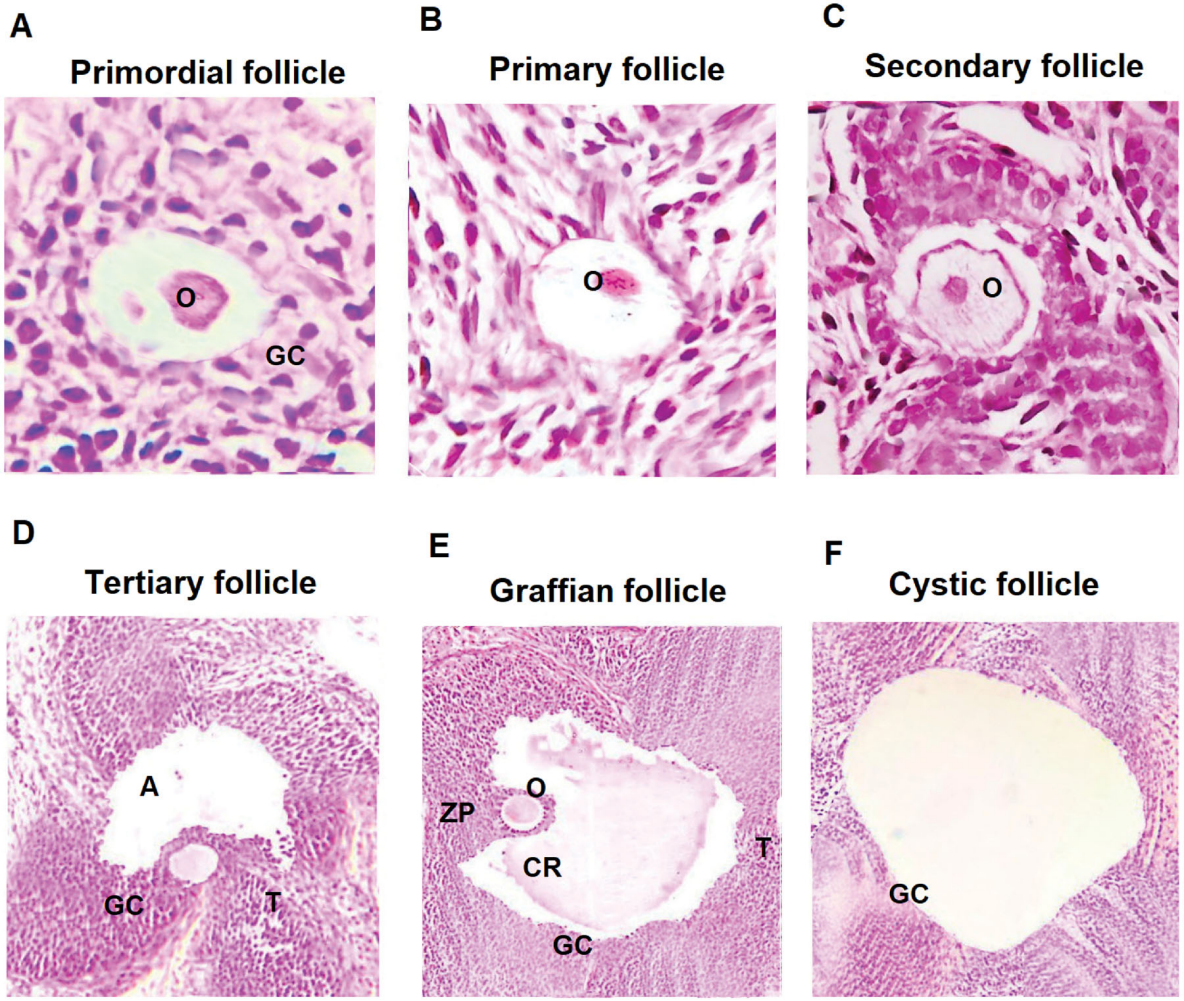
Histological analysis of various ovarian follicles in PCOS induced and control rats. (A) Primordial follicle showing flat layer of granulosa cells (GC) along with oocytes (O) at ×40. (B) Primary follicles with well-defined oocyte ×40. (C) Secondary follicle having fluid and defined oocyte ×40. (D) Tertiary follicle having theca (T), granulosa cells (GC) and antrum (A) ×40. (E) Graffian follicle having a huge antrum, oocyte, corona radiate (CR) and zona pellucida (ZP) ×40. (F) Cystic follicle showing a huge antrum devoid of oocyte. All images were captured at ×40.

The thickness analysis of thecal layer of secondary follicles and of granulosa in PCOS induced rats showed marked increase compared to control group, the APG and MTF treated rats showed a significant decrease in thickness compared PCOS vehicle treated rats. On analysing the wideness of granulosa layer of the tertiary follicles the PCOS control group showed a significant decrease compared to sham rats. The APG and MTF treated rats ameliorated the wideness values to normal levels, however the MTF group showed better results (*p* < .001) then the APG treated (*p* < .05). The MTF treatment in PCOS rats showed significant decrease in thickness of theca of the follicular cysts compared to PCOS vehicle treated group (*p* < .001). The treatment of APG also showed significant improvement of thickness of both theca and granulosa (*p* < .05) (Table 2; Figure 1).

### 3.5. Effect of Apigenin on number of primordial and primary ovarian follicles

The PCOS induced vehicle treated rats showed decrease in number of ovarian follicles (primordial and primary) compared to control rats. However, treatment of APG and MTF reversed the same significantly (*p* < .05). It was observed that the number of tertiary, secondary and Graffian follicles decreased significantly in PCOS vehicle treated rats compared to sham operated rats, however treatment of APG and MTF showed a significant increase in number of follicles. In addition to this a notable increase in cystic and atretic follicles (*p* < .001) associated with decreased corpus luteum was observed in PCOS vehicle treated rats which was reversed by treatment of APG and MTF (*p* < .001) suggesting recovery of the condition (Table 3; Figure 1).

**Figure 2.**
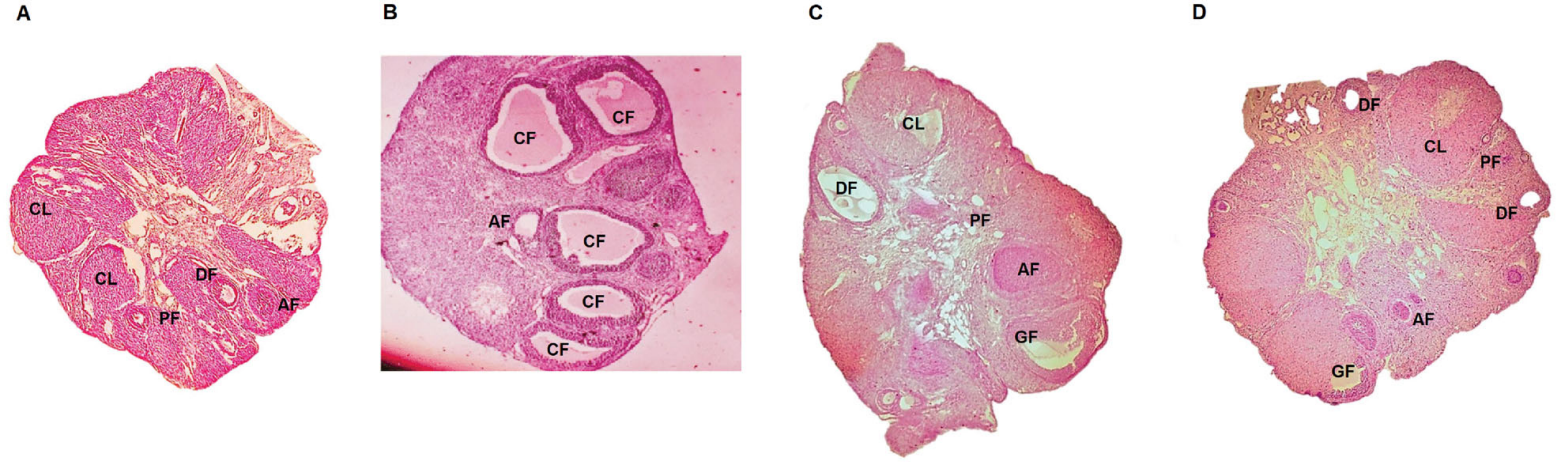
Cross sections of ovaries of rats of PCOS, control, metformin and Apigenin treated groups. (A) Cross section of ovarian tissue of control rats showing ovarian follicles along with primary follicles. (B) Ovarian tissues of PCOS rats showing various follicles. (C) Ovaries of MTF treated rats. (D) Ovaries of Apigenin treated rats.

### 3.6. Effect of Apigenin on antioxidant activity

A significant decrease in CAT, SOD, POD and GR activity was observed in the ovaries of PCOS induced vehicle treated rats (*p* < .001) compared to Sham operated control rats. The treatment of APG and MTF improved the levels of these antioxidant enzymes. With TBARS levels it was found that the levels were on significantly higher side in PCOS rats compared to sham group suggesting lipid peroxidation due to PCOS. Treatment of APG and MTF significantly decreased the levels of TBARS in PCOS rats (*p* < .01) (Table 4).

**Table 4.** Biochemical parameters.

Parameters	Control	PCOS	PCOS + MTF	PCOS + Apigenin
Oestradiol (pg/ml)	9.32 ± 0.42	1.63 ± 0.30***	9.26 ± 0.60^###^	8.87 ± 0.23^###^
Progesterone (ng/ml)	40.01 ± 2.31	11.16 ± 1.21***	33.95 ± 2.15^###^	35.01 ± 1.47^###^
Testosterone (ng/ml)	0.39 ± 0.004	1.68 ± 0.19***	0.53 ± 0.13^###^	0.81 ± 0.15^###^
Cholesterol (mg/dl)	55.65 ± 0.65	64.02 ± 1.02***	55.96 ± 1.52^###^	55.85 ± 1.52^###^
Triglycerides (mg/dl)	52.12 ± 0.85	68.25 ± 1.65***	58.96 ± 2.3^###^	56.45 ± 0.96^###^
HDL-C (mg/dl)	28.65 ± 0.23	22.20 ± 1.35***	26.20 ± 0.35*^###^	24.21 ± 0.85***
LDL-C (mg/dl)	16.67 ± 0.65	27.12 ± 1.85***	20.50 ± 1.21^#^	19.76 ± 1.36^##^
VLDL-C (mg/dl)	10.65 ± 0.35	14.21 ± 1.66*	11.86 ± 0.32	9.98 ± 0.35^#^
LDL-C/HDL-C	0.61 ± 0.003	1.65 ± 0.004***	0.73 ± 0.02*^###^	0.66 ± 0.004^###^
TC/HDL-C	1.98 ± 0.003	3.37 ± 0.04**	2.21 ± 0.04^##^	1.73 ± 0.003^###^
TG/HDL-C	1.85 ± 0.003	2.73 ± 0.45	2.35 ± 0.07	2.18 ± 0.20
Total protein (mg/g)	33.75 ± 0.55	27.35 ± 1.41*	31.75 ± 1.65	30.75 ± 1.01
CAT (u/mg)	33.68 ± 1.45	5.28 ± 0.15***	30.75 ± 0.88^###^	40.85 ± 0.95**^###@@@^
SOD (u/mg)	8.20 ± 0.20	5.61 ± 0.23***	6.86 ± 0.15**^#^	9.25 ± 0.33**^###@@@^
POD (µM/min)	0.52 ± 0.03	1.31 ± 0.11***	1.02 ± 0.05***^#^	0.76 ± 0.03*^###@@^
TBARS (µM/mg)	24.02 ± 0.35	32.56 ± 2.55**	31.52 ± 0.86*	26.25 ± 1.35^#^
GR (nM/mg protein)	1.45 ± 0.04	1.22 ± 0.08***	1.29 ± 0.03^##^	1.42 ± 0.02^##^

Values are mean ± SEM, *p < .05, **p < .01, ***p < .001 compared to control, #<.05, ##*p* < .01, ###*p* < .001 compared to PCOS group, @@*p* < .01, @@@*p* < .001 compared to PCOS + MTF group.

### 3.7. Effect of Apigenin in biochemical parameters

The PCOS induced rats showed a significantly increased levels of glucose, triglycerides and cholesterol (*p* < .001) compared to PCOS vehicle treated rats. However, the treatment of APG and MTF significantly decreased the levels of glucose, triglycerides and cholesterol (*p* < .001) against the vehicle treated rats (Tables 1 and 4).

When the levels of very low-density lipoprotein (VLDL) and low-density lipoprotein-cholesterol (LDL-C) were studied, it was found that the levels were significantly increased in PCOS vehicle treated rats (*p* < .001), whereas, the levels of High-density lipoprotein-cholesterol (HDL-C) were lowered in PCOS compared to sham control rats. Treatment of APG and MTF decreased VLDL-C and LDL-C and increased HDL-C levels compared to vehicle treated PCOS rats (Table 4). Parallel to the above results, a significant increase in ratio of LDL/HDL-C, TG/HDL-C and TC/HDL-C was observed in PCOS vehicle treated rats, whereas treatment of MTF and APG significantly improved the values (Table 4). The analysis of hormonal levels such as oestradiol and progesterone in PCOS rats showed that the levels were significantly decreased whereas the levels of testosterone were increased in PCOS rats compared to sham rats. However, treatment of APG and MTF ameliorated the levels of these hormones significantly compared to vehicle treated PCOS rats (Table 4).

### 3.8. Effect of Apigenin on levels of TNF-α and IL-6

**Figure 3.**
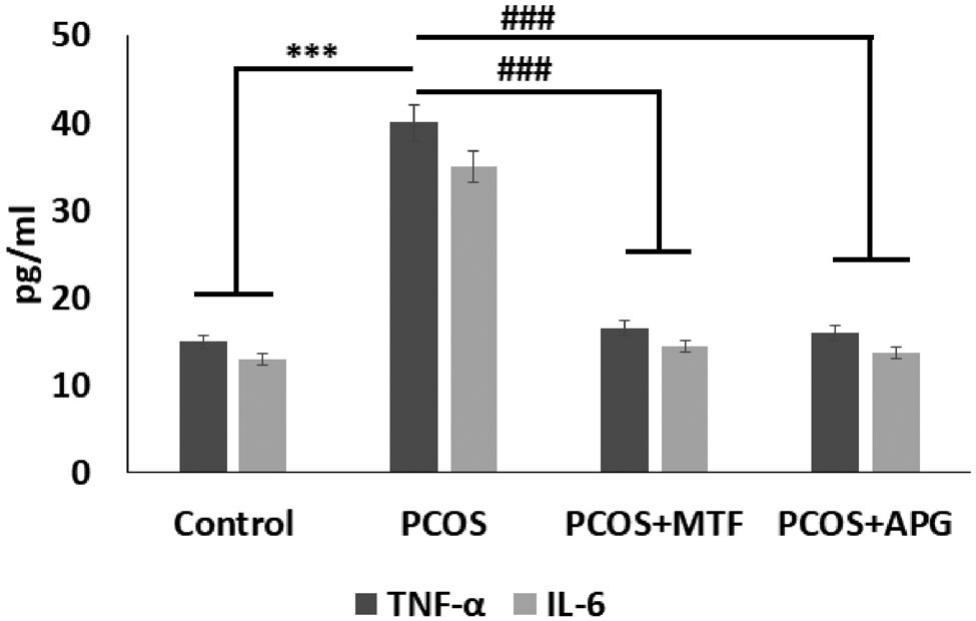
Elisa analysis of inflammatory cytokines in different treatment groups. ***p < .001 compared to control, ###*p* < .001 compared to PCOS group.

The PCOS rats showed significantly increased levels of Tumour Necrosis factor-α (TNF-α) and Interlukin-6 (IL-6) compared to sham operated control rats, suggesting involvement of inflammatory response in PCOS condition. The treatment of APG and MTF caused a significant suppression in levels of both the inflammatory markers compared to vehicle treated PCOS rats, thus confirming anti-inflammatory activity of APG (Figure 3).

## 4. Discussion

Present work successfully demonstrated the development of DHEA induced rat model of PCOS as described by Kim et al. [31]. The DHEA induced rat model has been used widely for studies involving PCOS [35,36]. DHEA is the first androgen which appears in adolescent among females [35]. We here opted the DHEA induced rat model as described by Kim et al. [31] and developed it successfully which was marked by increased body weight along with disturbed oestrous cycle. Treatment of Apigenin in PCOS rats showed significant drop in body weight which was in agreement to activity of Apigenin to regulate the obesity related genes [37]. Apigenin is a flavonoid which have been reported to correct fructose induced metabolic syndrome in rats [38]. Reports have suggested that signalling pathways associated with insulin and inflammation are linked with insulin resistance in poly cystic ovarian syndrome [39]. Apigenin regulates glucose metabolism in type-2 diabetic rats by regulating oxidative stress and inflammation [40]. Apigenin was found to ameliorate the beta-cell proliferation and biosynthesis of insulin in human pancreatic islets [41].

In addition to hyperglycaemia and inflammatory pathways oxidative stress is also one of the factors responsible for PCOS [42]. Excessive production of reactive oxygen species (ROS) which creates an imbalance in normal cells by affecting the indigenous anti-oxidant defence is termed as oxidative stress [43]. This disturbance due to ROS leads to imbalances in anti-oxidant enzyme which is evidenced by decreased SOD, CAT, POD, NADPH and GR activity [43]. Here, we found that PCOS rats showed increased oxidative stress as evidenced by decreased SOD, CAT, GR, NADPH and POD activity. The treatment of APG caused improvement in activity of SOD, CAT, POD, NADPH and GR the findings conformed to anti-oxidant activity of APG [44].

Thiobarbituric acid reactive substances (TBARS) is marker indicating lipid peroxidation [45]. Lipid peroxidation is confirmed to be the process of oxidative deprivation of lipids which triggers a free radical chain reaction in the lipids present in the membranes specifically the polyunsaturated fatty acids [46]. Dyslipidaemia is one of the main causes associated with coronary artery disease in subjects with PCOS [24,47]. In the present study increased TBRAS formation was observed in PCOS rats whereas treatment of APG resulted in suppression of these levels, confirming the anti-oxidant activity of APG [44]. Also, it has been reported earlier that Apigenin significantly reduced the levels of TC, TG and LDL-C followed by regulation of HDL-C in hyperlipidaemic rats, our findings were in agreement to this [30].

The hormonal study in DHEA induced PCOS rats suggested involvement of hyper-androgenized state for abnormal ovarian physiology [26]. Destruction of hypothalamus pituitary functioning results in elevation of both LH and testosterone hormones leading to diseased state in females [48,49]. The LH triggers the secretion of testosterone in the follicular thecal layer *via* the PI3K/Akt pathway [50]. The LH mediated phosphorylation of Akt in follicles is mediated by PI3K pathway leading to increased activity of 17α hydrolase enzyme, which is the key mediator for catalysing the steroidogenic conversion of progesterone to androgens leading to increased expression of androgens [51,52]. Our results showed that Apigenin treatment ameliorated the hormone pattern by preserving the levels of oestrogen, testosterone and progesterone.

The inflammatory mediators TNF-α and IL-6 are reported to involved in inflammatory conditions specifically in PCOS condition. Both TNF-α and IL-6 are found to overexpressed in adipose tissues of PCOS rats [53]. The levels of TNF-α and IL-6 both were found to suppressed in groups treated with flavonoid resveratrol [53]. In the present study we evidenced that the expression of both TNF-α and IL-6 was overexpressed in PCOS rats, however the treatment of MTF and Apigenin both resulted in suppression of TNF-α and IL-6. The findings of our study were in agreement to the study involving effect of resveratrol in PCOS [53].

Histological study helps in assessing the ovarian changes due to PCOS. In the present study the ovaries of PCOS induced rats showed presence of number of large cysts with decreased oocyte, hyperplasia of granulosa and theca layer along with enhanced atresia of follicles, the features were in agreement to earlier reports [54]. The corpus luteum is reported to play crucial role in secretion of progesterone which is responsible for regulating the reproductive cycles and also prepares the uterus for conception [55]. The reduction in number of tertiary and secondary follicles results in overproduction of androgens which obstructs the maturation process of follicles [54]. Contrarily the treatment of Apigenin and MTF in PCOS rats showed significant recovery of ovarian tissues which was evidenced by significant decrease in formation of cysts, regularity of luteinization and development of antral follicles [54]. It was also observed that treatment resulted in prominent antrum devoid of any cellular debris. The PCOS rats receiving treatment of Apigenin and MTF showed proliferation of number of healthy follicles in the ovarian cortex associated with improved vascularisation of thecal layer. The treated rats also showed increased corpus lutea suggesting renovation of oestrous cycle and normal functioning [54–56]. More studies are required for evaluating the molecular mechanisms associated with the beneficial effect of this useful flavonoid in PCOS.

In conclusion, the findings of our study suggested that Apigenin ameliorates the endocrine and metabolic comorbidities associated with PCOS condition. Apigenin, exerted beneficial effects on hormonal levels, lipid profile, body weight and glucose levels in PCOS rats. The flavonoid Apigenin also reduced oxidative stress, increased number of healthy follicles and helped in regaining the pleura of follicles. The treatment also inhibited the levels of important inflammatory cytokines TNF-α and IL-6 and confirmed involvement of inflammatory mediators. The study confirmed anti-androgen and ovary restoration potential which could be very beneficial in in treating PCOS condition. Clinical studies are needed so that Apigenin can be utilised as an adjuvant therapy alone or in combination with available therapeutic agents for treating PCOS.

## Data Availability

Data will be made available on reasonable request to corresponding author.
